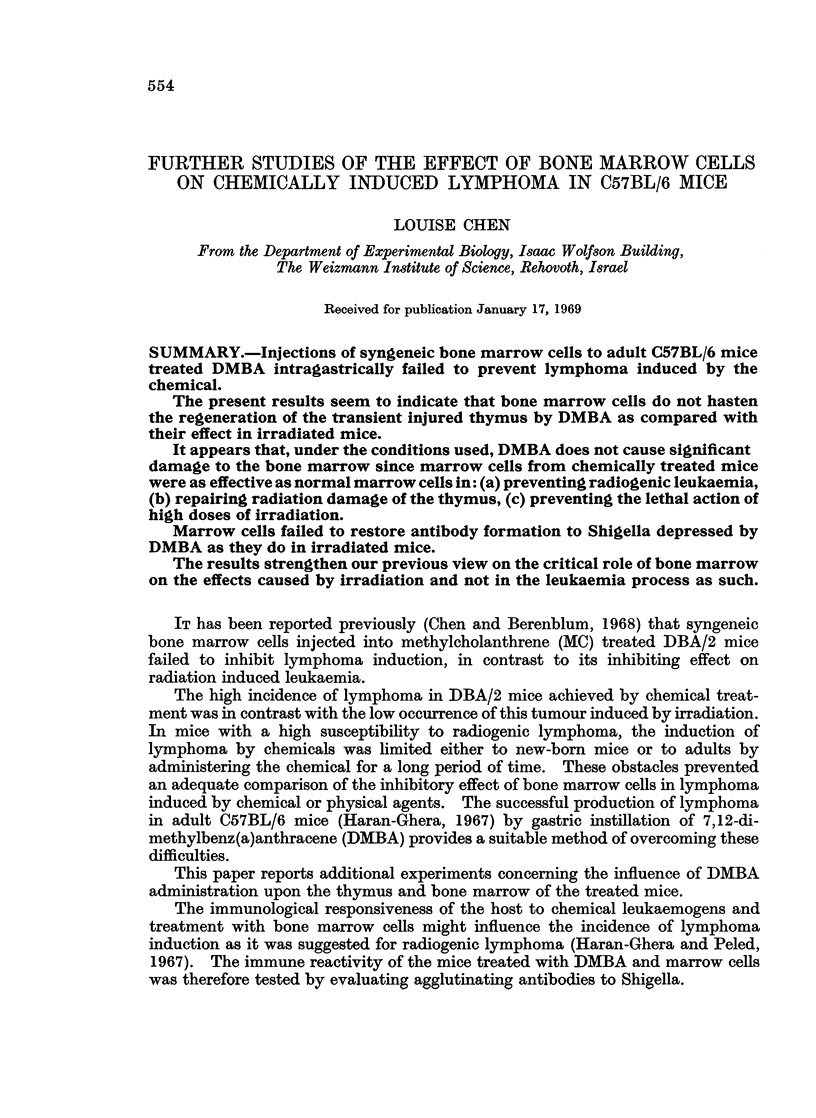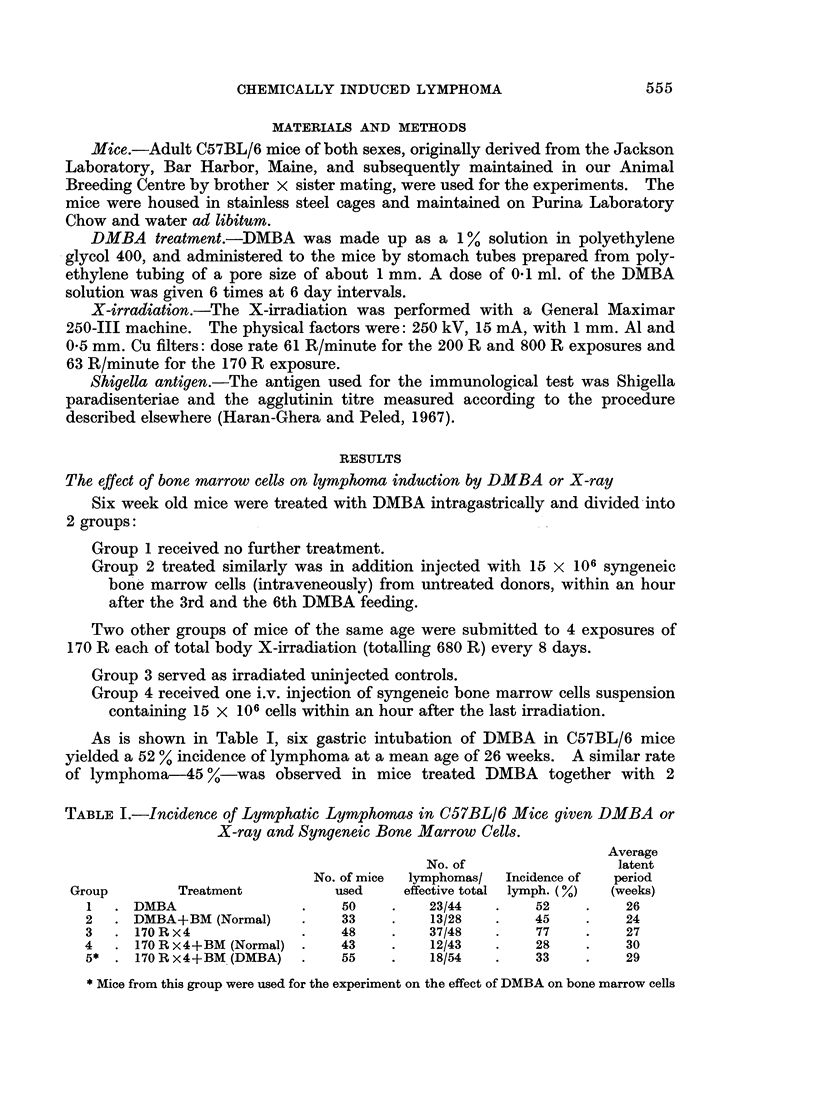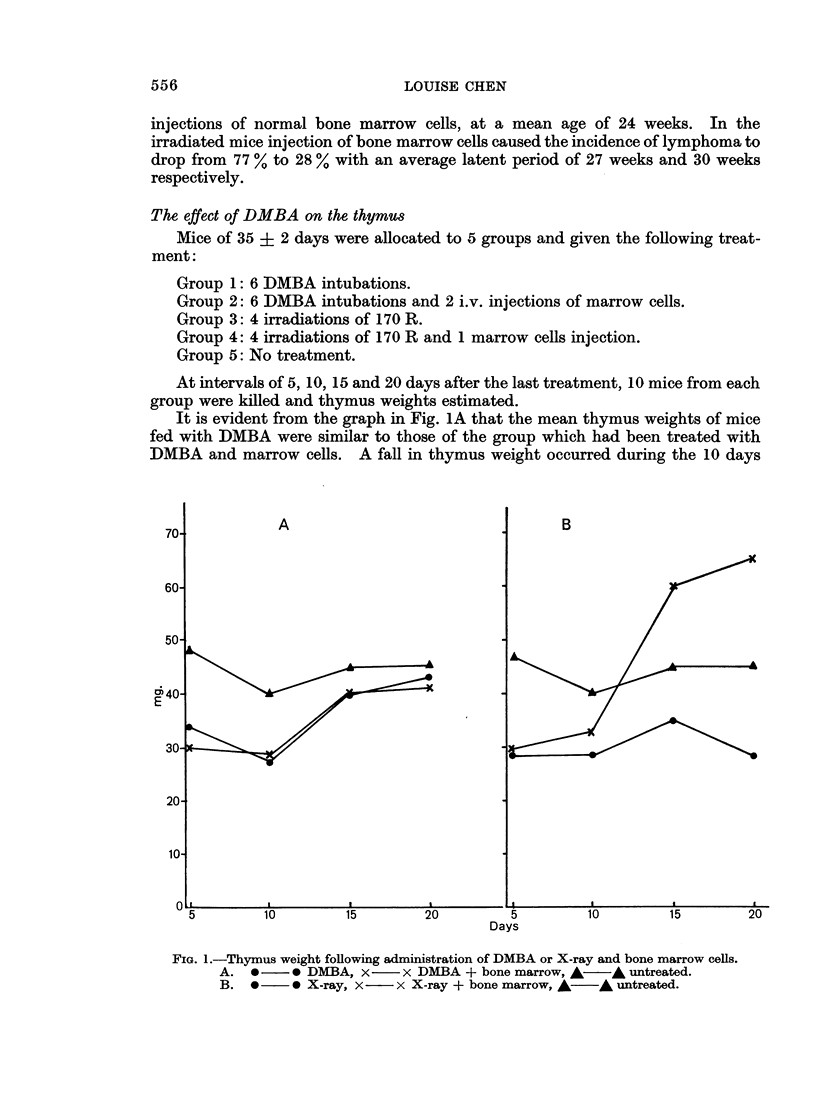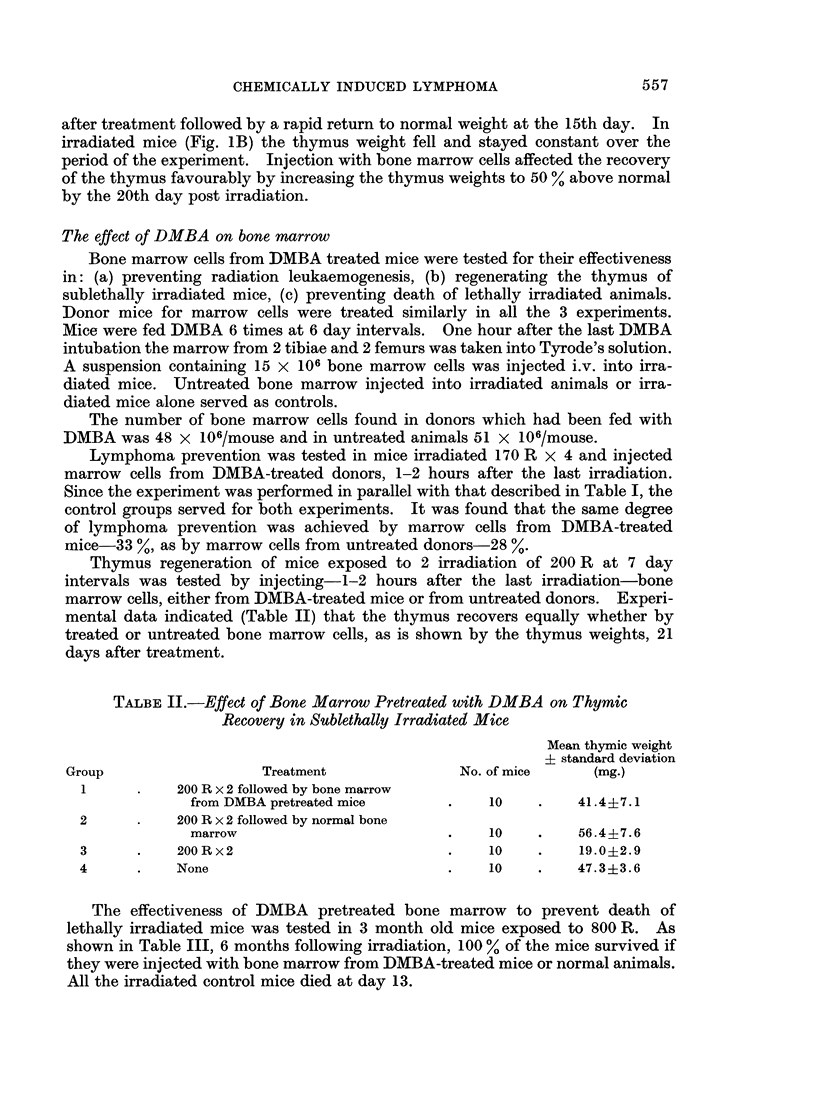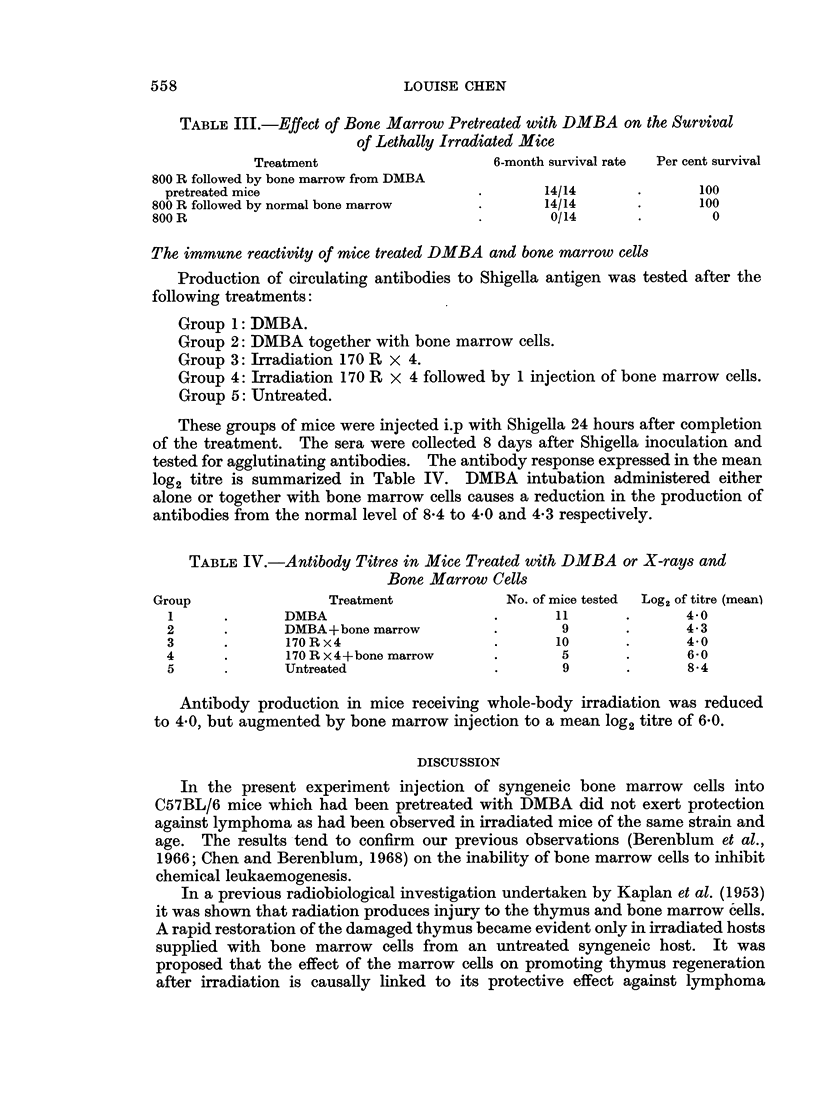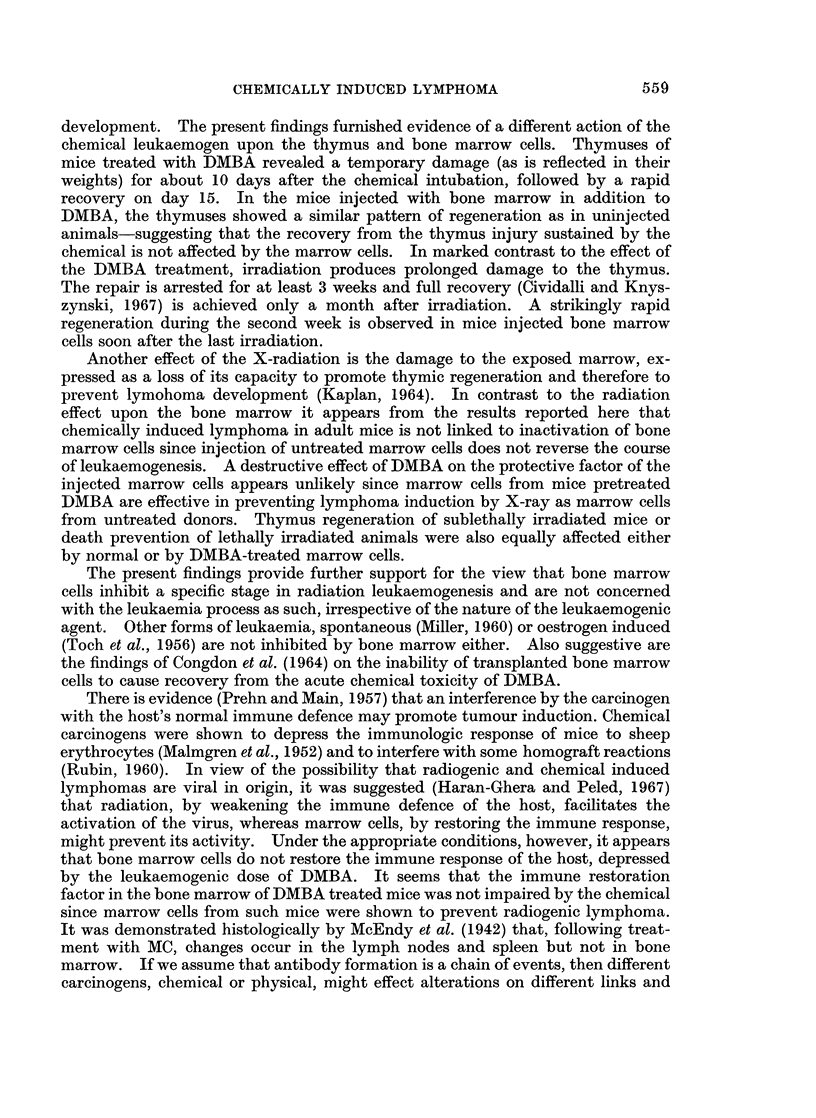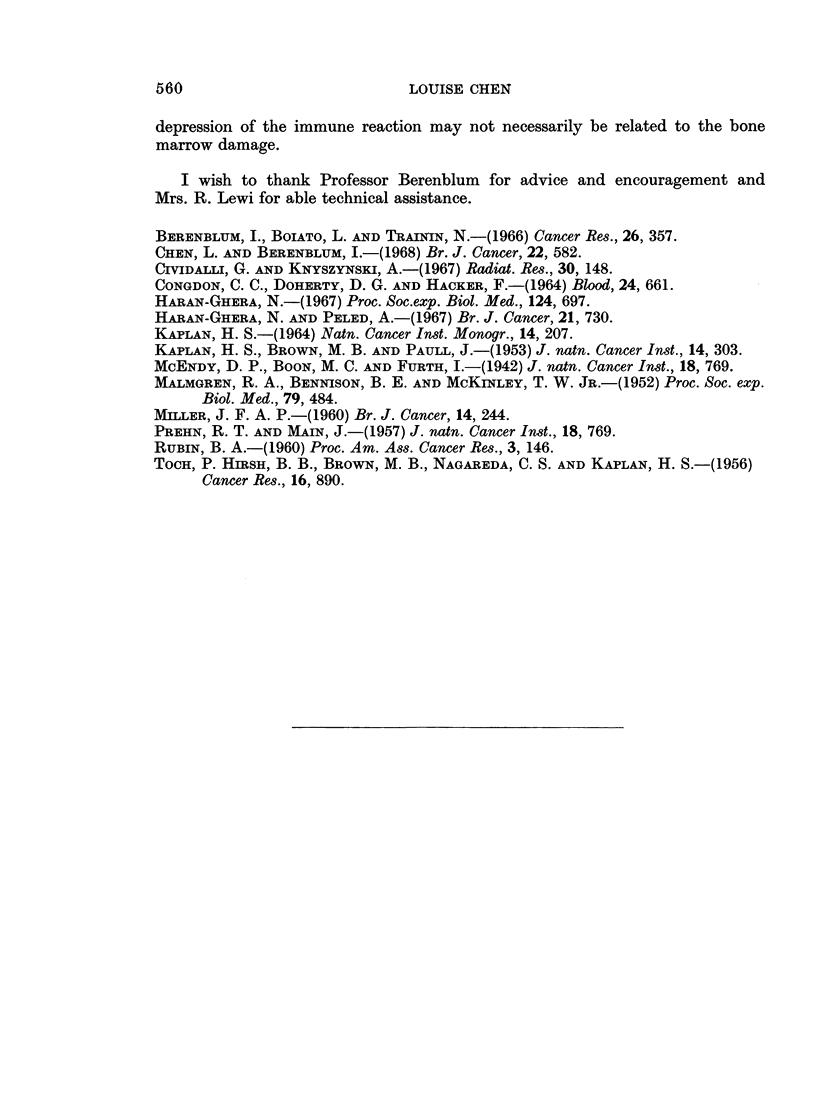# Further Studies of the Effect of Bone Marrow Cells on Chemically Induced Lymphoma in C57BL/6 Mice

**DOI:** 10.1038/bjc.1970.67

**Published:** 1970-09

**Authors:** Louise Chen

## Abstract

Injection of syngeneic bone marrow cell to adult C57BL/6 mice treated DMBA intragastrically failed to prevent lymphoma induced by the chemical.

The present result seem to indicate that bone marrow cells do not hasten the regeneration of the transient injuried thymus by DMBA as compared with their effect in irradiated mice.

It appears that, under the conditions used, DMBA does not cause significant damage to the bone marrow since marrow cells from chemically treated mice were as effective as normal marrow cells in: (a) preventing radiogenic leukaemia, (b) repairing radiation damage of the thymus, (c) preventing the lethal action of high doses of irradiation.

Marrow cells failed to restore antibody formation to Shigella depressed by DMBA as they do in irradiated mice.

The result strengthen our previous view on the critical role of bone marrow on the effects caused by irradiation and not in the leukaemia process as such.


### FURTHER STUDIES OF THE EFFECT OF BONE MARROW CELLS ON CHEMICALLY INDUCED LYMPHOMA IN C57BL/6 MICE

LOUISE CHEN

*From the Department of Experimental Biology, Isaac Wolfson Building, The Weizmann Institute of Science, Rehovoth, Israel*

Received for publication January 17, 1969

**SUMMARY.—Injections of syngeneic bone marrow cells to adult C57BL/6 mice treated DMBA intragastrically failed to prevent lymphoma induced by the chemical.**

**The present results seem to indicate that bone marrow cells do not hasten the regeneration of the transient injured thymus by DMBA as compared with their effect in irradiated mice.**

**It appears that, under the conditions used, DMBA does not cause significant damage to the bone marrow since marrow cells from chemically treated mice were as effective as normal marrow cells in: (a) preventing radiogenic leukaemia, (b) repairing radiation damage of the thymus, (c) preventing the lethal action of high doses of irradiation.**

**Marrow cells failed to restore antibody formation to Shigella depressed by DMBA as they do in irradiated mice.**

**The results strengthen our previous view on the critical role of bone marrow on the effects caused by irradiation and not in the leukaemia process as such.**

IT has been reported previously (Chen and Berenblum, 1968) that syngeneic bone marrow cells injected into methylcholanthrene (MC) treated DBA/2 mice failed to inhibit lymphoma induction, in contrast to its inhibiting effect on radiation induced leukaemia.

The high incidence of lymphoma in DBA/2 mice achieved by chemical treatment was in contrast with the low occurrence of this tumour induced by irradiation. In mice with a high susceptibility to radiogenic lymphoma, the induction of lymphoma by chemicals was limited either to new-born mice or to adults by administering the chemical for a long period of time. These obstacles prevented an adequate comparison of the inhibitory effect of bone marrow cells in lymphoma induced by chemical or physical agents. The successful production of lymphoma in adult C57BL/6 mice (Haran-Ghera, 1967) by gastric instillation of 7,12-dimethylbenz(a)anthracene (DMBA) provides a suitable method of overcoming these difficulties.

This paper reports additional experiments concerning the influence of DMBA administration upon the thymus and bone marrow of the treated mice.

The immunological responsiveness of the host to chemical leukaemogens and treatment with bone marrow cells might influence the incidence of lymphoma induction as it was suggested for radiogenic lymphoma (Haran-Ghera and Peled, 1967). The immune reactivity of the mice treated with DMBA and marrow cells was therefore tested by evaluating agglutinating antibodies to Shigella.

### MATERIALS AND METHODS

*Mice.*—Adult C57BL/6 mice of both sexes, originally derived from the Jackson Laboratory, Bar Harbor, Maine, and subsequently maintained in our Animal Breeding Centre by brother × sister mating, were used for the experiments. The mice were housed in stainless steel cages and maintained on Purina Laboratory Chow and water *ad libitum.*

*DMBA treatment.*—DMBA was made up as a 1% solution in polyethylene glycol 400, and administered to the mice by stomach tubes prepared from polyethylene tubing of a pore size of about 1 mm. A dose of 0·1 ml. of the DMBA solution was given 6 times at 6 day intervals.

*X-irradiation.*—The X-irradiation was performed with a General Maximar 250-III machine. The physical factors were: 250 kV, 15 mA, with 1 mm. Al and 0·5 mm. Cu filters: dose rate 61 R/minute for the 200 R and 800 R exposures and 63 R/minute for the 170 R exposure.

*Shigella antigen.*—The antigen used for the immunological test was Shigella paradisenteriae and the agglutinin titre measured according to the procedure described elsewhere (Haran-Ghera and Peled, 1967).

### RESULTS

### *The effect of bone marrow cells on lymphoma induction by DMBA or X-ray*

Six week old mice were treated with DMBA intragastrically and divided into 2 groups:

Group 1 received no further treatment.
Group 2 treated similarly was in addition injected with 15 × $10^6$ syngeneic bone marrow cells (intraveneously) from untreated donors, within an hour after the 3rd and the 6th DMBA feeding.

Two other groups of mice of the same age were submitted to 4 exposures of 170 R each of total body X-irradiation (totalling 680 R) every 8 days.

Group 3 served as irradiated uninjected controls.
Group 4 received one i.v. injection of syngeneic bone marrow cells suspension containing 15 × $10^6$ cells within an hour after the last irradiation.

As is shown in Table I, six gastric intubation of DMBA in C57BL/6 mice yielded a 52 % incidence of lymphoma at a mean age of 26 weeks. A similar rate of lymphoma—45 %—was observed in mice treated DMBA together with 2

TABLE I.—*Incidence of Lymphatic Lymphomas in C57BL/6 Mice given DMBA or X-ray and Syngeneic Bone Marrow Cells.*

| Group | Treatment | No. of mice used | No. of lymphomas/ effective total | Incidence of lymph. (%) | Average latent period (weeks) |
|---|---|---|---|---|---|
| 1 | DMBA | 50 | 23/44 | 52 | 26 |
| 2 | DMBA+BM (Normal) | 33 | 13/28 | 45 | 24 |
| 3 | 170 R×4 | 48 | 37/48 | 77 | 27 |
| 4 | 170 R×4+BM (Normal) | 43 | 12/43 | 28 | 30 |
| 5* | 170 R×4+BM (DMBA) | 55 | 18/54 | 33 | 29 |

* Mice from this group were used for the experiment on the effect of DMBA on bone marrow cells injections of normal bone marrow cells, at a mean age of 24 weeks. In the irradiated mice injection of bone marrow cells caused the incidence of lymphoma to drop from 77 % to 28 % with an average latent period of 27 weeks and 30 weeks respectively.

*The effect of DMBA on the thymus*

Mice of 35 ± 2 days were allocated to 5 groups and given the following treatment:

Group 1: 6 DMBA intubations.

Group 2: 6 DMBA intubations and 2 i.v. injections of marrow cells.

Group 3: 4 irradiations of 170 R.

Group 4: 4 irradiations of 170 R and 1 marrow cells injection.

Group 5: No treatment.

At intervals of 5, 10, 15 and 20 days after the last treatment, 10 mice from each group were killed and thymus weights estimated.

It is evident from the graph in Fig. 1A that the mean thymus weights of mice fed with DMBA were similar to those of the group which had been treated with DMBA and marrow cells. A fall in thymus weight occurred during the 10 days

FIG. 1.—Thymus weight following administration of DMBA or X-ray and bone marrow cells.

A. ●——● DMBA, ×——× DMBA + bone marrow, ▲——▲ untreated.

B. ●——● X-ray, ×——× X-ray + bone marrow, ▲——▲ untreated.

after treatment followed by a rapid return to normal weight at the 15th day. In irradiated mice (Fig. 1B) the thymus weight fell and stayed constant over the period of the experiment. Injection with bone marrow cells affected the recovery of the thymus favourably by increasing the thymus weights to 50 % above normal by the 20th day post irradiation.

*The effect of DMBA on bone marrow*

Bone marrow cells from DMBA treated mice were tested for their effectiveness in: (a) preventing radiation leukaemogenesis, (b) regenerating the thymus of sublethally irradiated mice, (c) preventing death of lethally irradiated animals. Donor mice for marrow cells were treated similarly in all the 3 experiments. Mice were fed DMBA 6 times at 6 day intervals. One hour after the last DMBA intubation the marrow from 2 tibiae and 2 femurs was taken into Tyrode's solution. A suspension containing $15 \times 10^6$ bone marrow cells was injected i.v. into irradiated mice. Untreated bone marrow injected into irradiated animals or irradiated mice alone served as controls.

The number of bone marrow cells found in donors which had been fed with DMBA was $48 \times 10^6$/mouse and in untreated animals $51 \times 10^6$/mouse.

Lymphoma prevention was tested in mice irradiated 170 R × 4 and injected marrow cells from DMBA-treated donors, 1–2 hours after the last irradiation. Since the experiment was performed in parallel with that described in Table I, the control groups served for both experiments. It was found that the same degree of lymphoma prevention was achieved by marrow cells from DMBA-treated mice—33 %, as by marrow cells from untreated donors—28 %.

Thymus regeneration of mice exposed to 2 irradiation of 200 R at 7 day intervals was tested by injecting—1–2 hours after the last irradiation—bone marrow cells, either from DMBA-treated mice or from untreated donors. Experimental data indicated (Table II) that the thymus recovers equally whether by treated or untreated bone marrow cells, as is shown by the thymus weights, 21 days after treatment.

TALBE II.—*Effect of Bone Marrow Pretreated with DMBA on Thymic Recovery in Sublethally Irradiated Mice*

| Group | Treatment | No. of mice | Mean thymic weight ± standard deviation (mg.) |
|---|---|---|---|
| 1 | 200 R × 2 followed by bone marrow from DMBA pretreated mice | 10 | 41.4±7.1 |
| 2 | 200 R × 2 followed by normal bone marrow | 10 | 56.4±7.6 |
| 3 | 200 R × 2 | 10 | 19.0±2.9 |
| 4 | None | 10 | 47.3±3.6 |

The effectiveness of DMBA pretreated bone marrow to prevent death of lethally irradiated mice was tested in 3 month old mice exposed to 800 R. As shown in Table III, 6 months following irradiation, 100 % of the mice survived if they were injected with bone marrow from DMBA-treated mice or normal animals. All the irradiated control mice died at day 13.

TABLE III.—*Effect of Bone Marrow Pretreated with DMBA on the Survival of Lethally Irradiated Mice*

| Treatment | 6-month survival rate | Per cent survival |
|---|---|---|
| 800 R followed by bone marrow from DMBA pretreated mice | 14/14 | 100 |
| 800 R followed by normal bone marrow | 14/14 | 100 |
| 800 R | 0/14 | 0 |

*The immune reactivity of mice treated DMBA and bone marrow cells*

Production of circulating antibodies to Shigella antigen was tested after the following treatments:

Group 1: DMBA.
Group 2: DMBA together with bone marrow cells.
Group 3: Irradiation 170 R × 4.
Group 4: Irradiation 170 R × 4 followed by 1 injection of bone marrow cells.
Group 5: Untreated.

These groups of mice were injected i.p with Shigella 24 hours after completion of the treatment. The sera were collected 8 days after Shigella inoculation and tested for agglutinating antibodies. The antibody response expressed in the mean $\log_2$ titre is summarized in Table IV. DMBA intubation administered either alone or together with bone marrow cells causes a reduction in the production of antibodies from the normal level of 8·4 to 4·0 and 4·3 respectively.

TABLE IV.—*Antibody Titres in Mice Treated with DMBA or X-rays and Bone Marrow Cells*

| Group | Treatment | No. of mice tested | $\text{Log}_2$ of titre (mean) |
|---|---|---|---|
| 1 | DMBA | 11 | 4·0 |
| 2 | DMBA+bone marrow | 9 | 4·3 |
| 3 | 170 R×4 | 10 | 4·0 |
| 4 | 170 R×4+bone marrow | 5 | 6·0 |
| 5 | Untreated | 9 | 8·4 |

Antibody production in mice receiving whole-body irradiation was reduced to 4·0, but augmented by bone marrow injection to a mean $\log_2$ titre of 6·0.

### DISCUSSION

In the present experiment injection of syngeneic bone marrow cells into C57BL/6 mice which had been pretreated with DMBA did not exert protection against lymphoma as had been observed in irradiated mice of the same strain and age. The results tend to confirm our previous observations (Berenblum *et al.*, 1966; Chen and Berenblum, 1968) on the inability of bone marrow cells to inhibit chemical leukaemogenesis.

In a previous radiobiological investigation undertaken by Kaplan *et al.* (1953) it was shown that radiation produces injury to the thymus and bone marrow cells. A rapid restoration of the damaged thymus became evident only in irradiated hosts supplied with bone marrow cells from an untreated syngeneic host. It was proposed that the effect of the marrow cells on promoting thymus regeneration after irradiation is causally linked to its protective effect against lymphoma development. The present findings furnished evidence of a different action of the chemical leukaemogen upon the thymus and bone marrow cells. Thymuses of mice treated with DMBA revealed a temporary damage (as is reflected in their weights) for about 10 days after the chemical intubation, followed by a rapid recovery on day 15. In the mice injected with bone marrow in addition to DMBA, the thymuses showed a similar pattern of regeneration as in uninjected animals—suggesting that the recovery from the thymus injury sustained by the chemical is not affected by the marrow cells. In marked contrast to the effect of the DMBA treatment, irradiation produces prolonged damage to the thymus. The repair is arrested for at least 3 weeks and full recovery (Cividalli and Knyszynski, 1967) is achieved only a month after irradiation. A strikingly rapid regeneration during the second week is observed in mice injected bone marrow cells soon after the last irradiation.

Another effect of the X-radiation is the damage to the exposed marrow, expressed as a loss of its capacity to promote thymic regeneration and therefore to prevent lymohoma development (Kaplan, 1964). In contrast to the radiation effect upon the bone marrow it appears from the results reported here that chemically induced lymphoma in adult mice is not linked to inactivation of bone marrow cells since injection of untreated marrow cells does not reverse the course of leukaemogenesis. A destructive effect of DMBA on the protective factor of the injected marrow cells appears unlikely since marrow cells from mice pretreated DMBA are effective in preventing lymphoma induction by X-ray as marrow cells from untreated donors. Thymus regeneration of sublethally irradiated mice or death prevention of lethally irradiated animals were also equally affected either by normal or by DMBA-treated marrow cells.

The present findings provide further support for the view that bone marrow cells inhibit a specific stage in radiation leukaemogenesis and are not concerned with the leukaemia process as such, irrespective of the nature of the leukaemogenic agent. Other forms of leukaemia, spontaneous (Miller, 1960) or oestrogen induced (Toch *et al.*, 1956) are not inhibited by bone marrow either. Also suggestive are the findings of Congdon *et al.* (1964) on the inability of transplanted bone marrow cells to cause recovery from the acute chemical toxicity of DMBA.

There is evidence (Prehn and Main, 1957) that an interference by the carcinogen with the host's normal immune defence may promote tumour induction. Chemical carcinogens were shown to depress the immunologic response of mice to sheep erythrocytes (Malmgren *et al.*, 1952) and to interfere with some homograft reactions (Rubin, 1960). In view of the possibility that radiogenic and chemical induced lymphomas are viral in origin, it was suggested (Haran-Ghera and Peled, 1967) that radiation, by weakening the immune defence of the host, facilitates the activation of the virus, whereas marrow cells, by restoring the immune response, might prevent its activity. Under the appropriate conditions, however, it appears that bone marrow cells do not restore the immune response of the host, depressed by the leukaemogenic dose of DMBA. It seems that the immune restoration factor in the bone marrow of DMBA treated mice was not impaired by the chemical since marrow cells from such mice were shown to prevent radiogenic lymphoma. It was demonstrated histologically by McEndy *et al.* (1942) that, following treatment with MC, changes occur in the lymph nodes and spleen but not in bone marrow. If we assume that antibody formation is a chain of events, then different carcinogens, chemical or physical, might effect alterations on different links and depression of the immune reaction may not necessarily be related to the bone marrow damage.

I wish to thank Professor Berenblum for advice and encouragement and Mrs. R. Lewi for able technical assistance.

Berenblum, I., Boiato, L. and Trainin, N.—(1966) *Cancer Res.*, **26**, 357.
Chen, L. and Berenblum, I.—(1968) *Br. J. Cancer*, **22**, 582.
Cividalli, G. and Knyszynski, A.—(1967) *Radiat. Res.*, **30**, 148.
Congdon, C. C., Doherty, D. G. and Hacker, F.—(1964) *Blood*, **24**, 661.
Haran-Ghera, N.—(1967) *Proc. Soc.exp. Biol. Med.*, **124**, 697.
Haran-Ghera, N. and Peled, A.—(1967) *Br. J. Cancer*, **21**, 730.
Kaplan, H. S.—(1964) *Natn. Cancer Inst. Monogr.*, **14**, 207.
Kaplan, H. S., Brown, M. B. and Paull, J.—(1953) *J. natn. Cancer Inst.*, **14**, 303.
McEndy, D. P., Boon, M. C. and Furth, I.—(1942) *J. natn. Cancer Inst.*, **18**, 769.
Malmgren, R. A., Bennison, B. E. and McKinley, T. W. Jr.—(1952) *Proc. Soc. exp. Biol. Med.*, **79**, 484.
Miller, J. F. A. P.—(1960) *Br. J. Cancer*, **14**, 244.
Prehn, R. T. and Main, J.—(1957) *J. natn. Cancer Inst.*, **18**, 769.
Rubin, B. A.—(1960) *Proc. Am. Ass. Cancer Res.*, **3**, 146.
Toch, P. Hirsh, B. B., Brown, M. B., Nagareda, C. S. and Kaplan, H. S.—(1956) *Cancer Res.*, **16**, 890.